# Water Decontamination from Cr(VI) by Transparent Silica Xerogel Monolith

**DOI:** 10.3390/ijms24087430

**Published:** 2023-04-18

**Authors:** Marco Zannotti, Andrea Rossi, Marco Minicucci, Stefano Ferraro, Laura Petetta, Rita Giovannetti

**Affiliations:** 1 Chemistry Interdisciplinary Project, School of Science and Technology, Chemistry Division, University of Camerino, 62032 Camerino, Italy; andrea.rossi@unicam.it (A.R.); stefano.ferraro@unicam.it (S.F.);; 2 School of Science and Technology, Physics Division, University of Camerino, 62032 Camerino, Italy; marco.minicucci@unicam.it

**Keywords:** chromium, environmental remediation, silica xerogel, absorption kinetics, equilibrium study

## Abstract

Cr(VI) is highly soluble and mobile in water solution and extremely toxic. In order to obtain a specific material with adsorption properties towards Cr(VI), and that can be used in environmental remediation of water contaminated with Cr(VI), one-step sol-gel technique, at low temperature (50 °C), has been optimized to prepare transparent silica-based xerogel monolith by using tetraethyl orthosilicate as precursor. The obtained xerogel, with disk shape, was fully characterized by Raman, BET, FE-SEM and XRD analysis. The results indicated that the material showed silica amorphous phase and high porosity. The study of the adsorption properties towards different concentrations of Cr(VI), in the form of HCrO_4_^−^ in acidic condition, showed prominent results. The absorption kinetics were evaluated by studying different models, the final result showing that the absorption of Cr(VI) occurred through intra-particle diffusion process, following two steps, and that the absorption equilibrium is regulated by Freundlich isotherm model. The material can be restored by reducing the hazardous Cr(VI) to Cr(III), a less toxic form of chromium, by 1,5-diphenylcarbazide, and with successive treatment in acidic water.

## 1. Introduction

Nowadays, chromium pollution of water and soil is a serious environmental problem; after lead, chromium is the second most common inorganic pollutant discharged in the environment [1].

Chromium is used in different industrial processes such as leather tanning, electroplating, textile dyeing and plastic productions, with the generations of hazardous waste containing relatively high amounts of chromium. In the environment, chromium exists as Cr(III) and Cr(VI), and, with respect to the trivalent form, the hexavalent chromium is 100 times more toxic, carcinogenic and mutagenic; direct exposition to Cr(VI) causes eyes irritation, allergic reactions, asthma, and lung and kidney cancer [2,3,4].

Cr(VI) is highly soluble and mobile in water solution and can be present in different species, depending on its concentration and the pH of the aqueous environment. Chromate ions (CrO_4_^2−^) exist only at basic pH, the hydrogen chromate ion (HCrO_4_^−^) between acidic pH 1 and neutral pH 7, while the acidic form of Cr(VI)—the chromic acid (H_2_CrO_4_)—at a very acidic pH of less than 1. The dichromate species (Cr_2_O_7_^2−^) can be produced in solution when the concentration of Chromium is higher than 1 g L^−1^. These features with the strong oxidant ability of Cr(VI) compounds make chromium pollution even more dangerous in water solutions [2,5].

Several methods such as reduction to Cr(III) and precipitation, chemical extraction, dialysis, electrochemical separation, coagulation and ion-exchange have been applied to reduce the Cr(VI) concentration and its effect [6,7,8,9,10].

Adsorption method is probably the most effective, with low-cost easy operation and being environmentally friendly; the adsorption process applied to Cr(VI)-contaminated water shows high removal efficiency, low energy cost and chemical investment, and the possibility to reuse the absorbent material [11,12,13]. Mesoporous silica-based materials for their unique and tunable physicochemical properties such as great chemical, mechanical and thermal stability with a large surface area, are ideal candidates as novel adsorbent materials [2,14]. In particular, silica-based xerogel is a material with high porosity, than can be produced by sol-gel synthesis through hydrolysis and condensation of alkoxysilanes (Si(OR)_4_) precursor, obtained in the presence of acid or base as catalyst. In this case, in contrast to aerogel, the formed gel is then dried under atmospheric conditions to remove the liquid and to produce a xerogel with the desired shape [15,16]. The molar ratio between alkoxides/water/solvent, pH and temperature, influence the different polymeric structures such as linear, entangled chain, clusters and colloidal particles [17].

Xerogels obtained by conventional drying have high density due to shrinkage during drying normally and are affected by cracking and shrinkage; to overcome this problem, drying control additives are added on the precursor solution [18,19]. The transparency of the material makes it suitable for optical devices and spectroscopic investigations amounts and furthermore the high degree of silanol groups gives high porosity to the materials and available binding groups for chemical absorptions or interaction with inorganic or organic species.

In this work, a silica-based xerogel transparent monolith was produced by a simple one-step sol-gel technique at very low temperatures (50 °C), with tetraethyl orthosilicate (TEOS) as a precursor. The obtained monolithic disk was deeply characterized by Raman, BET, FE-SEM and XRD analysis and used as an adsorbent for Cr(VI) in the form of HCrO_4_^−^. The material was used as prepared, without modification and with no addition of other organic compounds; its complete transparency permitted to monitor the adsorption by easy operation, such as UV-Vis spectroscopy. The absorption of Cr(VI) was monitored during time, at different concentrations, in order to define the adsorption properties and to obtain equilibrium and kinetic results. The Cr(VI) can be converted to Cr(III) by a treatment with 1,5-diphenylcarbazide and subsequently the xerogel restored by washing in acid solution.

## 2. Results and Discussion

### 2.1. Silica-Based Xerogel Monolith

**Figure 1:**
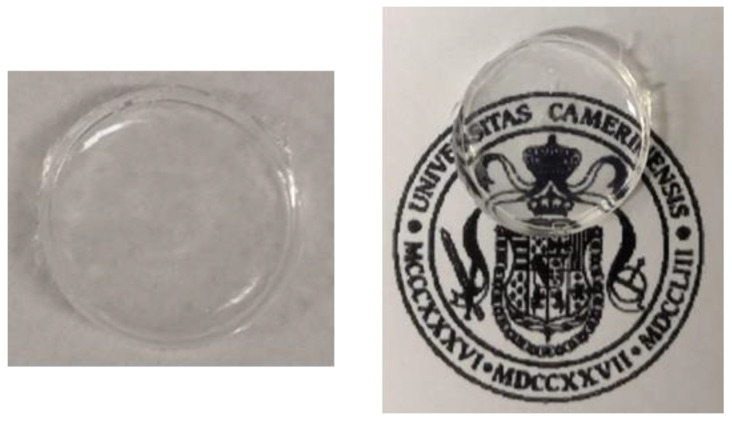
Silica-based xerogel monolith obtained by sol-gel method.

The silica-xerogel produced by sol-gel method at low temperature, produced a uniform and transparent disk as reported in Figure 1.

In this procedure, the addition of DMF, as drying control chemical additive (DCCA), permitted to obtain a crack-free monolith. Despite that the procedure involved HCl, DMF decreased the acidity of the precursor solution by acting as hydrogen bond acceptor, promoting thereby the hydrolysis rate of TEOS. In this case, the formation of hydrogen bonds of DMF with silanol groups facilitated the removal of water molecules preventing the interaction of them with the silanol groups [20]. At the same time, the nucleophilicity of the silanol groups of TEOS is enhanced, with the result of faster condensation. Furthermore, DMF can hydrolyze with the formation of formic acid promoting the subsequent decrease of pH during sol-gel process. It is possible to say that initially there is a faster acid-catalyzed process, and later a based catalyzed process, that lead to a high interconnectivity of small oligomers with a decrease of the gelation time [20]. The addition of DMF, a polar aprotic solvent, by hydrogen bonding to the silanol groups, inhibits the coalescence of the silica particles. The final result is a material without crack, but with larger pore size; the capillary forces in this case will be weaker and thus the stress exerted on the gel during drying will be smaller.

### 2.2. Silica-Based Xerogel Characterization

**Figure 2:**
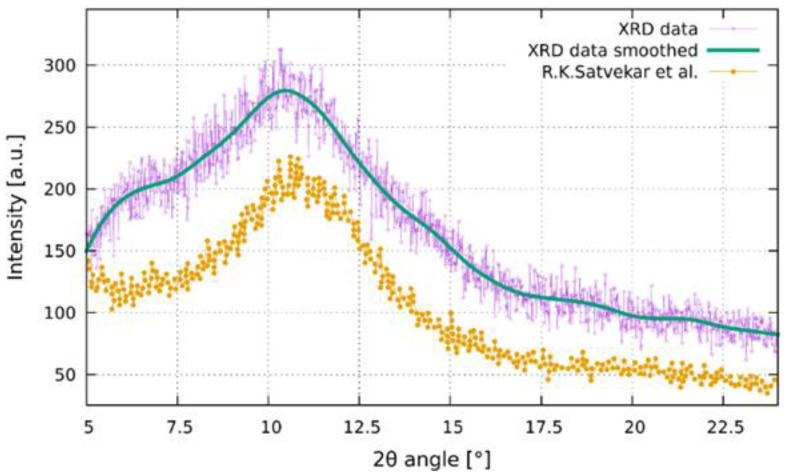
X-ray power diffraction pattern of silica-based xerogel [21].

The silica-based xerogel was characterized by XRD analysis, as reported in Figure 2. The dried material after two hours at 70 °C showed a broadened peak in the 2θ range of 8–15°; the feature corresponds to the amorphous silica matrix [21]. The results indicated that no crystalline phase was formed during the initial drying of thin films prepared with TEOS at low temperature.

**Figure 3:**
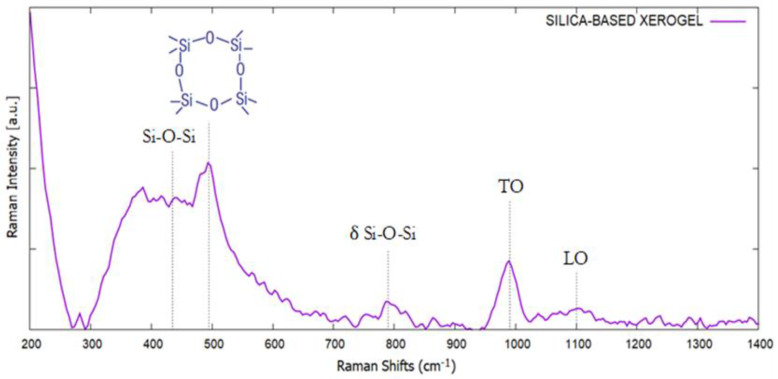
Raman spectrum of silica-based xerogel.

The Raman spectrum of silica-based xerogel, reported in Figure 3, shows the typical bands of amorphous SiO_2_. The broadened peak at 440 cm^−1^, denoted as R-band, is attributed to the coupled stretching–bending mode of the Si-O-Si bridges. The sharp peak at 495 cm^−1^ is assigned to the symmetric stretching modes of four-membered of SiO_2_ rings and usually is called as D_1_ peak. The band at 790 cm^−1^ is related to bending modes of the Si-O-Si inter-tetrahedral angle, while the bands at 995 and 1110 cm^−1^ are related to the transversal optic (TO) and longitudinal optic (LO) asymmetric stretching modes of Si-O [22,23,24,25]. 

**Figure 4:**
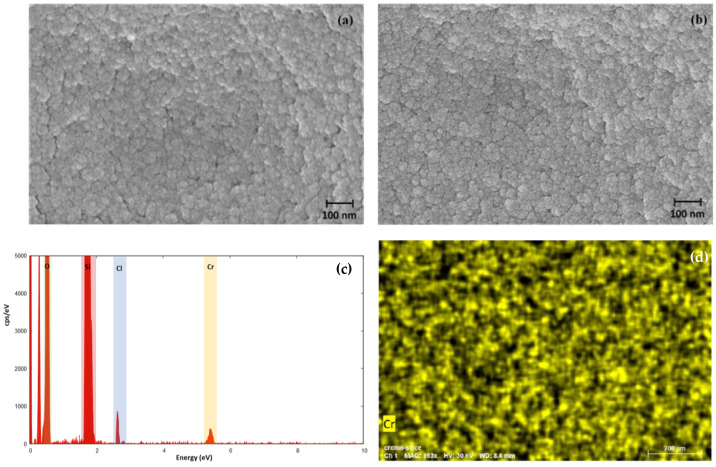
SEM images of silica-based xerogel (**a**) before and (**b**) after the absorption of Cr(VI), (**c**) EDX spectrum of the silica-based xerogel after the adsorption of Cr(VI) and (**d**) map distribution image for Cr.

The morphology of the silica-based xerogel was evaluated by SEM microscopy as reported in Figure 4a where the SEM image shows a monodisperse sample with uniform spherical particles. Figure 4b reports the SEM image of the xerogel after the absorption of Cr(VI); the morphology and dimensions of particles remains the same, to prove that the material is not altered after the absorption of HCrO_4_^−^ on the silica matrix. The EDX spectrum reported in Figure 4c confirms the adsorption of Cr(VI) by the detection of the peak at 5.41 eV, while the map distribution image shows an homogeneous distribution of Cr on the sample.

**Figure 5:**
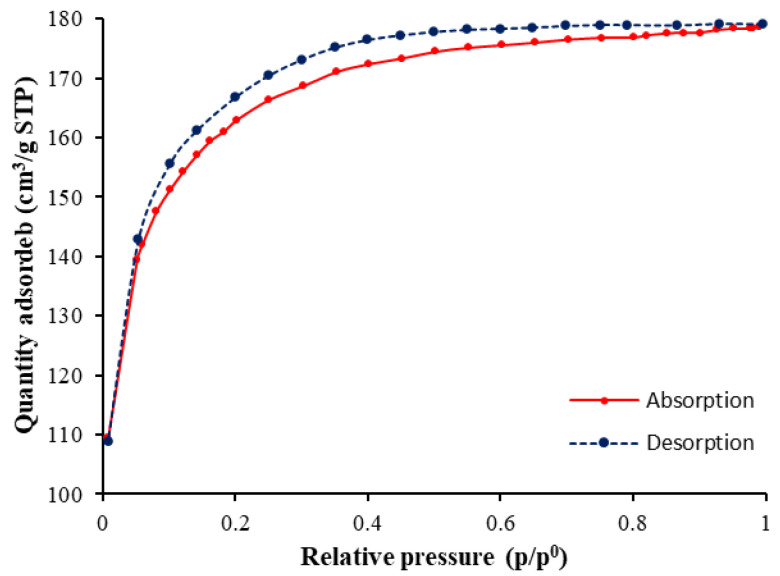
N_2_ gas adsorption–desorption isotherm for the silica-based xerogel.

BET gas adsorption indicates that the silica xerogel is porous with the size of the pores at around 2.38 nm and specific surface are of 547.33 m^2^/g. Figure 5 shows the N_2_ gas adsorption with a hysteresis loop during the desorption, the latter is common for porous materials like inorganic oxides and glasses [26].

### 2.3. Kinetic Studies

The kinetic in the absorption of solute onto a solid adsorbent surface can be describe by different kinetic models; as example a first order mechanism is showed in the Equation (1) [27]:

(1) $$\frac{dq_{t}}{dt}=k_{1}\left(q_{e}-q_{t}\right)$$

where *q_t_* is the adsorbate (mg/g), in this case HCrO_4_^−^, adsorbed on the xerogel at time *t* in mg/g, *q_e_* is the equilibrium absorption capacity of the absorbent at the equilibrium (mg/g), and *k*_1_ is the rate constant of the absorption process. 

The linear form of the Equation (1) is reported in the Equation (2) as follow:

(2) $$\ln\left(q_{e}-q_{t}\right)=lnq_{e}-k_{1}t$$

By plotting *t* vs. *ln(q_e_ − q_t_)*, the value of *k*_1_ can be determined; in this case, the constant is proportional to the starting concentration of the absorbate in solution. The absorption process takes place only on localized sites and no interaction between the absorbed solute are present; the absorption occurs as a monolayer on the surface of the absorbent material and the surface coverage does not affect the energy related to the absorption [28]. 

A second approach to absorption can be the pseudo second order kinetic, described by Equation (3):

(3) $$\frac{dq_{t}}{dt}=k_{2}{\left(q_{e}-q_{t}\right)}^{2}$$

and by the linear form of Equation (4):

(4) $$\frac{t}{q_{t}}=\frac{1}{k_{2}q_{e}^{2}}+\frac{t}{q_{e}}$$

where *k*_2_ is the pseudo-second order constant.

This model presumes the same conditions for the pseudo-firs order model except that the absorption rate is explained by a rate equation of order 2. The absorption rate depends on the solute amount on the absorbent surface, and the driving force (*q_e_ − q_t_*) is proportional to the active sites available on the absorbent material. In this case, by plotting *t/q_e_* vs. *t/q_t_*, the intercept on the x-axis represented by 1/*k*^2^*q_e_*^2^, permits to calculate the pseudo-second order kinetic constant, *k*_2_ [29].

Another kinetic model to explain the absorption process is that of Elovich, which initially was applied to the absorption of the gas molecule. The Elovich model permits the prediction of the mass and surface diffusion, activation and deactivation energy. In this case, the assumption is that the adsorption rate of the solute molecules decreases exponentially with the increasing amount of adsorbed solute. The model is described by the following Equations (5) and (6) [29]:

(5) $$\frac{dq_{t}}{dt}=\alpha exp^{-\beta q_{t}}$$

(6) $$q_{t}=\frac{1}{\beta }\ln\left[\alpha \beta \right]+\frac{1}{\beta }\ln t$$

where *α* is the initial absorption rate (mg/g min), *β* is the desorption constant. The plot of *q_t_* vs. *t* permits to calculate the parameter and evaluate the absorption on the heterogeneous surface of the absorbent. The Elovich model predicts that the absorption energy increases linearly with the surface coverage and interactions between the absorbed molecules on the surface of the material are possible [28].

The absorption of a solute can occur in several steps and the overall process can be controlled by different steps such as film or external diffusion, surface diffusion, pore diffusion and adsorption on the pore surface with a combination between them. This absorption model is defined as intra-particle diffusion and described by Equation (7) [30]

(7) $$q_{t}=k_{id}t^{1/2}+C$$

where *k_id_* is the intraparticle diffusion constant (mg/g half minute) and *C* indicates the thickness of the boundary layer (mg/g). Plotting *q_t_* vs. *t*^0.5^, the resultant plot is a straight line, proving that the absorption process is regulated by intraparticle diffusion; on the other hand, if the plot exhibits multi-linear plots, two or more steps regulate the absorption process [30].

**Figure 6:**
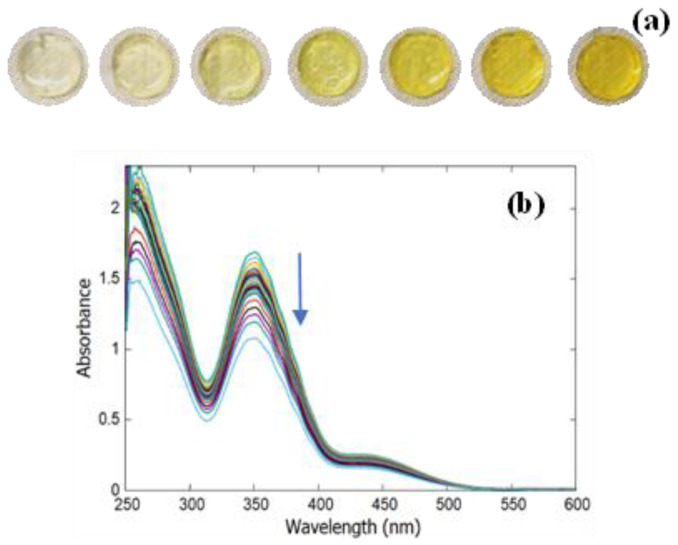
(**a**) Silica-based xerogel samples after absorption treatment by using different concentrations of Cr(VI) solutions from 36.53 to 269 mg/L, (**b**) UV-Vis spectra of Cr(VI) solution, with initial concentration of 55.41 mg/L, at different times during the absorption treatment with silica-based xerogel, the blue arrow represents the decrease in absorption during time.

In order to study the adsorption properties of silica xerogel monolith, different concentrations of Cr(VI) from 36.53 to 269 mg/L were tested in acidic condition; as evidenced in Figure 6a, after the Cr(VI) adsorption, the color of xerogel changed from colorless to yellow. The adsorption processes were spectrophotometrically analyzed, monitoring over time the decrease of the absorbance solution containing Cr(VI) (Figure 6b) with the aim of defining the appropriate kinetic models. 

The UV-Vis spectra reported in Figure 6b are typical of water HCrO_4_^−^ solution with the presence of the absorption band at 350 nm and a shoulder at around 445 nm; another intense peak is detected at 260–265 nm that is related to the O-Cr^6+^ charge transfer between oxygen and Cr(VI) of chromate ions in its tetrahedral structure [31,32]. During the adsorption process on the xerogel material, the UV-Vis spectrum of HCrO_4_^−^ decreases, confirming the uptake of Cr(VI) by the silica-based material; the obtained spectra maintain the same profile, demonstrating that the xerogel did not influence the spectrum of the aqueous solution and that therefore the Cr(VI) remains stable as HCrO_4_^−^ ion during the absorption process. The results of the kinetic study and the fitting with the different kinetic equation model are reported in Table 1. As it is possible to observe for the pseudo first order, the correlation factor is lower with respect to the other kinetic models; therefore, it is possible to confirm that, for the three studied concentrations, the absorption process is not described by pseudo-first order kinetic. On the other hand, for the pseudo-second order and the Elovich model, higher correlation factors were obtained, but, in this case, the fitting of the curve and the calculated *q_e_* did not match very well the experimental results. 

**Figure 7:**
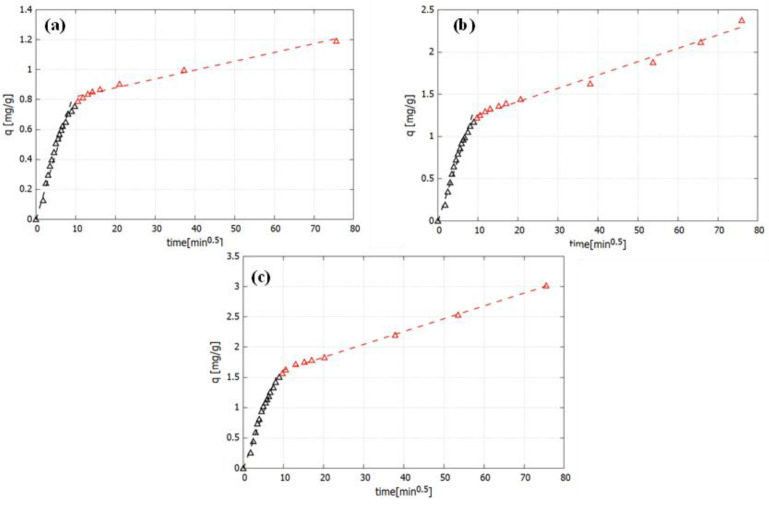
Intraparticle diffusion model of Cr(VI) adsorption on silica-based xerogel of (**a**) 36.53, (**b**) 55.41 and (**c**) 73.7 mg/L starting concentrations; black triangles: faster first absorption phase, red triangles: slower second absorption phase.

As it is possible to observe from Table 2 and Figure 7, the absorption process can be well described by the intraparticle diffusion model. In this case, the absorption process takes place in two absorption phases—a faster initial one, that for the lower concentration lasted 90 min, while it lasted 80 min for the other two higher concentrations of Cr(VI), followed by a slowed second phase. The absorption constants of the two phases for the three tested concentrations of Cr(VI) are reported in Table 2.

### 2.4. Absorption Equilibrium Study

The influence of the Cr(VI) concentration on the adsorption process has been evaluated by studying the equilibrium data with Freundlich and Langmuir isotherms, reported as Equations (8) and (9), respectively [33]:

(8) $$\ln Q_{e}=\ln K_{F}+\frac{1}{n}\ln C_{e}$$

(9) $$\frac{C_{e}}{Q_{e}}=\frac{1}{K_{L}}+\frac{a_{L}}{K_{L}}C_{e}$$

where *C_e_* is the Cr(VI) concentration (mg/L), *Q_e_* is the adsorbed amount on the monolith xerogel in mg/g, *K_F_* is the Freundlich constant that represent the absorption capacity and 1/*n* is the absorption intensity. For the Langmuir model, *K_L_* and *a_L_* are the Langmuir constants and the ratio between them represents the theoretical saturation capacity of the monolith xerogel. Table 3 reports the concentration of the starting solution and the relative absorbed amount of Cr(VI), *Q_e_*, evaluated for the absorption equilibrium study.

**Figure 8:**
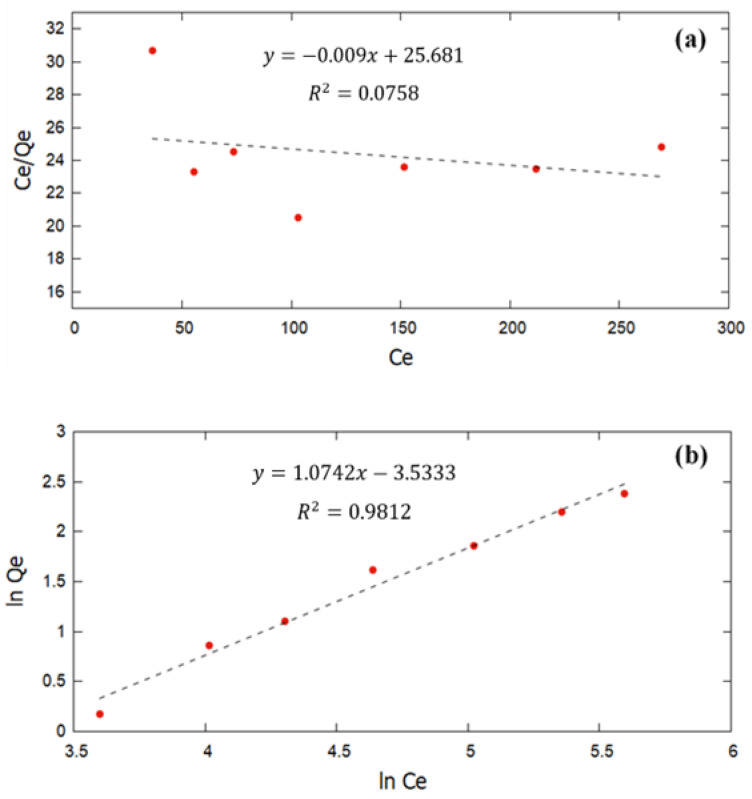
(**a**) Langmuir isotherm and (**b**) Freundlich isotherm plots.

Figure 8 reports the isotherm plots for the Langmuir and Freundlich model, respectively; as it is possible to observe that the absorption process follows a Freundlich model, with high correlation factor *R*^2^ confirming that the adsorption takes place as multiple layers on the porous surface of the silica-based xerogel monolith. Table 4 reports the equilibrium data for the Freundlich isotherm model.

**Figure 9:**
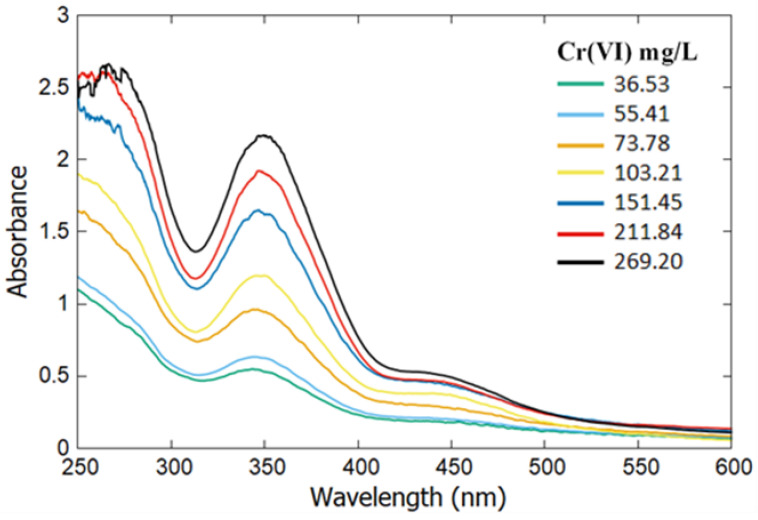
Absorption spectra of Cr(VI) adsorbed on silica-based xerogel at different initial concentrations (mg/L) from 36.53 to 269.20 mg/L.

The absorption spectrum of Cr(VI) absorbed on the silica-based xerogel monolith can be easily detected by fiber optic spectrophotometer as reported in Figure 9. The Cr(VI) is adsorbed as HCrO_4_^−^ and remains stable in this form after the absorption inside the silica matrix confirmed by the unchanged UV-Vis spectra, with respect to the liquid phase. In this case, the calculated molar extinction coefficient of HCrO_4_^−^ on the xerogel is 383.22 L·mol^−1^·cm^−1^. 

**Scheme 1:**
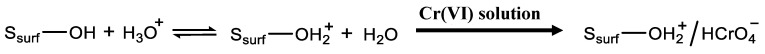
Absorption mechanism of Cr(VI) on silica-based xerogel in acidic pH condition.

In this case, the adsorption properties of silica in acidic conditions can be explained considering the high surface area and porosity that characterizes this adsorbent material and the presence of protonated hydroxyl groups on the silicates surface (S_surf_) that, during the adsorption process, can favor the adsorption of HCrO_4_^−^ ions [34] (Scheme 1). 

**Figure 10:**
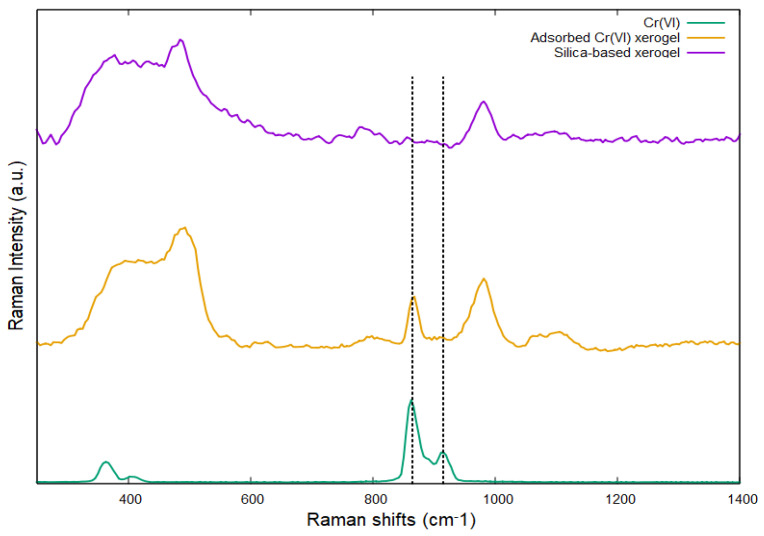
Raman spectra of silica-based xerogel before (purple) and after Cr(VI) absorption (yellow) and K_2_CrO_4_ powder (green).

Raman spectrum reported in Figure 10 also confirms the actual presence of Cr(VI) onto the matrix of the silica-based xerogel. In fact, the comparison between silica-based xerogel and adsorbed Cr(VI) xerogel, evidences the presence of a new peak at 860 cm^−1^ attributable to the Cr^VI^-O bonds, which is also confirmed by the Raman spectrum of Cr(VI) salt [35]. 

### 2.5. Material Regeneration

**Figure 11:**
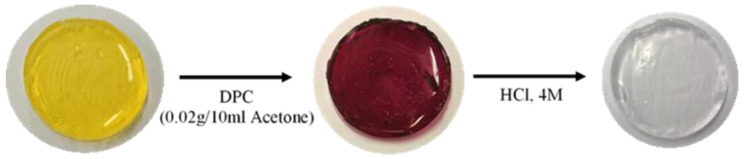
Regeneration method of silica-based xerogel.

The xerogel monolith was treated with 1,5-diphenylcarbazide (DPC) and, after this process, the Cr(VI), highly toxic and dangerous, was reduced directly on the material to the less toxic Cr(III). In this case, the DPC reduces the Cr(VI) to Cr(III), oxidizing itself to 1,5-diphenylcarbazone (DPCA), the latter form a purple complex with Cr(III), named as Cr(III)-DPCA [36]. To regenerate the material, the Cr(III)-DPCA xerogel was then treated with HCl (4M); in this case, as it is possible to observe in Figure 11, the xerogel returns transparent and can be reused for another cycle of absorption for Cr(VI). In Figure 11, the schematized method for the regeneration of the xerogel after Cr(VI) absorption treatment is reported.

**Figure 12:**
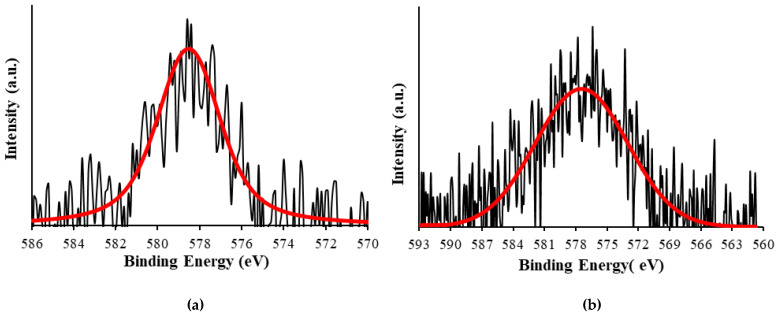
XPS spectra of Cr2p_3/2_ for silica-based xerogel (**a**) before and (**b**) after treatment with DPC.

To confirm the reduction of Cr(VI) to Cr(III), XPS analysis on dried and pulverized sample was performed; the results are reported in Figure 12.

The XPS spectra show the peak relative to Cr2p_3/2_; when Cr(VI) is absorbed on the silica-based xerogel, the signal is positioned at 578.6 eV, while after the reduction with DPC and the formation of the relative complex Cr(III)-DPCA, the XPS signal of chromium is at 577.4 eV, confirming the reduction to Cr(III) on the silica-based xerogel [37].

## 3. Materials and Methods

Hydrochloric acid (HCl), ethanol (EtOH), tetraethyl orthosilicate (TEOS), Dimethylformamide (DMF) and 1,5-Diphenylcarbazide are purchased from Merck and used without further purification.

### 3.1. Silica-Based Xerogel Preparation

Two separate solutions were prepared: the first contained HCl/H_2_O and the second was a mixture of TEOS/EtOH/DMF. The acidic aqueous solution was added to the precursor solution and stirred at room temperature for 1 h. The molar ratio between H_2_O/EtOH/TEOS/DMF was 16:4:1:2.5. Successively, 1 mL of the precursor solution was transferred into a plastic container covered with a plastic cap and left in the oven at 50 °C for seven days. Afterwards, a final treatment at 70 °C for 4 h was carried out obtaining a transparent xerogel monolith disk with a diameter of around 18 mm and a thickness of around 2.5 mm.

### 3.2. Xerogel Characterization

The xerogel crystalline structure was evaluated by X-ray diffraction (XRD) technique. The monolith xerogel was crushed into powder and analyzed by a Debye–Scherrer diffractometer equipped with an INEL CPS 180 (INEL, Artenay, France) curved position sensitive detector; in such a way, a drastic reduction of the acquisition time for each pattern was obtained. The X-rays source was a Mo K-alpha (lambda = 0.7093 Å). X-ray generated by a Philips sealed X-ray tube and monochromatized through a graphite crystal along the 002 plane. The sample was inserted in a glass capillary tube (diameter 100 μm) and centered on the beam.

In addition, the xerogel was characterized by Raman spectroscopy by using a micro-Raman spectrometer (iHR320, Horiba). The sample was analyzed with a green laser (532 nm), 50× objective outlet at room temperature. 

BET measurements were performed in which the xerogel was first dried at 80 °C for 24 h, after the porosity and the specific area were evaluated through volumetric N_2_ adsorption at 77 K using an ASAP 2020 (Micrometrics) instrument. The porosity of the materials was determined by BJH method (Halsey thickness equation).

The morphology of the silica-based xerogel was studied by using Field Emission Scanning Electron Microscopy (FE-SEM, Sigma 300, Zeiss, Germany) operated at 3 kV. The dried xerogel by using a self-adhesive carbon conductive tab was deposited on aluminum stabs. To prevent charging during the analysis, the sample was sputtered with chromium (5 nm) by Quorum Q150T-ES (Quorum Technologies, Lewes, UK). In order to perform EDX analysis, a portion of the adsorbed-Cr(VI) xerogel was sputtered with graphite.

### 3.3. Chromium Absorption Test and Kinetics Studies

The silica-based xerogel was tested for the absorption of Cr(VI) from water solutions. Different concentrations of K_2_CrO_4_ from 6.8 × 10^−4^ to 5 × 10^−3^ M at a pH of about 2 were prepared and studied in the absorption on silica-based xerogel. The absorption of Cr(VI) was evaluated at different times of absorption, monitoring the UV-Vis spectra of the Cr(VI) solutions by Cary 8454 Diode Array System spectrophotometer (Agilent Technologies, Santa Clara, CA, USA). The UV-Vis absorption spectra of silica-xerogel with the absorbed Cr(VI) were directly recorded by an HR4000-UV-NIR Ocean Optics fibre optic spectrophotometer.

The spectroscopic data were used for a detailed kinetics study and for the evaluation of the better isotherm model to explain the absorption of chromium on silica-based xerogel.

### 3.4. Material Regeneration

After the absorption of Cr(VI) the xerogel was treated with a solution of 1,5-diphenylcarbazide (DPC), prepared by dissolving 0.04 g of DPC in 10 mL of acetone. In this case, Cr(VI) was reduced to Cr(III), then the xerogel monolith was washed several time with HCl solution (4M). An X-ray Photoelectron analysis (XPS, VG Scientific Ltd., East Grinstead, UK) of the dried silica-xerogel, before and after the treatment with DPC, was performed. The deconvolution of the XPS spectrum was carried out by Fityk software (Microsoft, GitHub, San Francisco, CA, USA).

## 4. Conclusions

A silica-based transparent xerogel monolith, with a disk shape, was synthesized by an easy single step sol-gel process, at a low temperature around 50 °C, by using TEOS as silica precursor and without surface modification. The material was characterized by Raman, SEM, XRD and BET, showing the typical features of silica material with high porosity and amorphous phase. 

The synthetized xerogel was easy to handle and showed prominent absorption capacity towards Cr(VI), in the form of HCrO_4_^−^. The transparency of the material permitted an evaluation of the absorption properties directly at naked eyes on the material and an easy quantification by UV-Vis analysis. The absorption process was deeply investigated, resulting in an intraparticle diffusion kinetic model, that occurs in two steps, while the absorption equilibrium was regulated by Freundlich isotherm model. 

In addition, the absorbed Cr(VI) after reduction to Cr(III) by the use of DPC, can be successively regenerated by HCl washing. This silica material can be therefore advantageously applied on environmental remediation of water contaminated with Cr(VI).

## Figures and Tables

**Table 1:** Kinetic constants and correlation factors for Cr(VI) absorption, analyzed by pseudo-first and second order and by Elovich model.

Cr(VI) (mg/L).	*q_exp_* (mg/g)	Pseudo-First Order			Pseudo-Second Order			Elovich			
		$\ln(q_{e}-q_{t})=\ln q_{e}-k_{1}t$			$\frac{t}{q_{t}}=\frac{1}{k_{2}q_{e}^{2}}+\frac{t}{q_{e}}$			$q_{t}=\frac{1}{\beta }\ln\left[\alpha \beta \right]+\frac{1}{\beta }\ln t$			
		*k*_1_ (min^−1^)	*q_e_* (mg/g)	*R* ^2^	*k*_2_ (min^−1^)	*q_e_* (mg/g)	*R* ^2^	*q_e_* (mg/g)	*α* (mg/g min)	*β* (g/mg)	*R* ^2^
36.53	1.1903	0.0137	1.1903	0.9170	0.0146	1.1784	0.9980	1.3616	0.1605	6.3694	0.9603
55.41	2.3751	0.0102	2.3751	0.9078	0.0066	2.3493	0.9873	2.2835	0.3030	3.8610	0.9755
73.78	3.0160	0.0991	3.0160	0.8830	0.0034	2.9656	0.9895	2.9769	0.3804	3.0321	0.9824

**Table 2:** Kinetic constants and correlation factors of intraparticle diffusion model for Cr(VI) absorption by silica-based xerogel.

Cr(VI) (mg/L)	Intraparticle Diffusion					
	$q_{t}=k_{id}t^{1/2}+C$					
	Phase 1			Phase 2		
	*kd*_1_ (mg/g min^0.5^)	Duration (min)	*R* ^2^	*kd*_2_ (mg/g min^0.5^)	Duration (min)	*R* ^2^
36.53	0.0891	90	0.9712	0.0069	----	0.9879
55.41	0.1462	80	0.9734	0.0158	----	0.987
73.78	0.1745	80	0.9762	0.0212	----	0.9978

**Table 3:** Starting concentration and absorbed concentration at equilibrium of evaluated Cr(VI).

Cr(VI) (mg/L)	*Q_e_* (mg/g)
36.53	1.1903
55.41	2.3751
73.78	3.0160
103.21	5.4302
151.45	6.4223
211.84	9.0002
269.20	10.8608

**Table 4:** Equilibrium data and correlation factor of Freundlich isotherm model.

Freundlich Isotherm Model	
1/*n*	1.0742
*lnK_F_*	3.5333
*K_F_*	0.0292
*R* ^2^	0.9812

## Data Availability

Additional data is available on request.
